# Emergence of Novel *Anaplasma* Species in the Mediterranean Area

**DOI:** 10.3390/ani15071029

**Published:** 2025-04-02

**Authors:** Valentina Chisu, Rosanna Zobba, Giovanna Masala, Giovanna Chessa, Laura Giua, Piera Bianco, Carla Cacciotto, Emanuela Bazzoni, Alberto Alberti

**Affiliations:** 1 Istituto Zooprofilattico Sperimentale “G. Pegreffi” della Sardegna, Via Duca degli Abruzzi 8, 07100 Sassari, Italy; giovanna.masala@izs-sardegna.it (G.M.); giovanna.chessa@izs-sardegna.it (G.C.); laura.giua@izs-sardegna.it (L.G.); piera.bianco@izs-sardegna.it (P.B.); 2 Dipartimento di Medicina Veterinaria, Università degli Studi di Sassari, 07100 Sassari, Italy; ccacciotto@uniss.it (C.C.); e.bazzoni2@phd.uniss.it (E.B.); alberti@uniss.it (A.A.)

**Keywords:** *Candidatus* Anaplasma cinensis, *Candidatus* Anaplasma boleense, zoonosis, emerging infectious diseases, reservoirs of infection

## Abstract

The emergence of new *Anaplasma* strains in the Mediterranean region poses a challenge for both veterinary medicine and public health, as it can lead to more complex diagnostic and treatment strategies. Species related to *Anaplasma platys* and *A. phagocytophilum*, two important tick-borne pathogens, have been reported in several Mediterranean countries. However, the data on their presence in Sardinia remain limited. This study aimed to **identify the** *Anaplasma* **species genetically related to zoonotic agents,** such as *A. phagocytophilum* or *A. platys*, in **Sardinian horses, cattle, and swine.** Using various molecular approaches targeting the ***groEL*** and ***gltA*** genes, the results confirm the presence of *Candidatus* (*Ca.*) *Anaplasma turritanum* and reveal the emergence of *Candidatus* (*Ca.*) *Anaplasma cinensis* and an *A. phagocytophilum*-like strain, designated as *A. phagocytophilum*-like 2, in Sardinian horses and cattle. These findings underscore the importance of ongoing surveillance in Sardinia and similar Mediterranean regions, as well as the urgent need for enhanced diagnostic tools and preventive strategies. Given the zoonotic potential of these emerging strains, these findings emphasize the need for greater vigilance in both veterinary and human health sectors to mitigate the risks associated with tick-borne *Anaplasma* species transmission. Ongoing research and proactive measures are essential to reduce the public health burden and prevent the spread of these pathogens across animal and human populations.

## Simple Summary

Bacterial tick-borne diseases are a major concern for both human and animal health in subtropical, tropical, and Mediterranean regions. The island of Sardinia has been identified as a hotspot for tick-borne infections, particularly those caused by bacteria from the *Anaplasma* genus. The distribution and species composition of these bacteria are expanding globally, driven by factors such as climate change, human activity, animal movements, and the shifting distribution of tick vectors. This paper provides evidence for the emergence of novel *Anaplasma* candidate species and strains in the Mediterranean area and suggests a possible role for hosts as reservoirs of these infections. Given the overlap in clinical symptoms caused by different tick-borne pathogens, and the fact that only *Anaplasma phagocytophilum* is currently included in human and animal diagnostic tests, it is crucial to include these new *Anaplasma* species in the diagnostic routine for tick-borne infections. This will help develop effective control strategies based on a comprehensive One Health approach.

## 1. Introduction

Anaplasmosis is a tick-borne disease caused by the bacterial organisms of the genus *Anaplasma,* which affect a wide range of wild and domestic mammalian hosts, including humans [1]. Although *Anaplasma* species are primarily of veterinary importance, human cases have also been reported worldwide since 1994 [2,3,4,5,6]. Thanks to the molecular characterization of multiple target genes, *Anaplasma* species have been classified into six distinct species that infect various mammals and specific host cell types, such as monocytes (*Anaplasma bovis*), neutrophils (*Anaplasma phagocytophilum*), erythrocytes (*Anaplasma ovis*, *Anaplasma marginale* and *Anaplasma centrale*), and platelets (*Anaplasma platys*) [1,7,8]. In addition to these well-recognized *Anaplasma* species, several new strains with potential diagnostic and therapeutic relevance have recently been identified in a variety of hosts [7]. For example, *Anaplasma odocoilei* sp. nov. was isolated from the white-tailed deer (*Odocoileus virginianus*) in the United States [9], *Candidatus* Anaplasma boleense was initially identified in *Hyalomma asiaticum* ticks in China [10,11], and *Anaplasma capra* was initially isolated from asymptomatic goats in China and subsequently recognized as a novel tick-transmitted zoonotic pathogen [4,12,13,14]. *Candidatus Anaplasma cinensis* is a newly described species within the *Anaplasma* genus, closely related to *A. platys*, an established pathogen of both veterinary and public health concern [10,15].

Ixodid ticks are the primary vectors and reservoirs of *Anaplasma* species in nature, with species from the genera *Ixodes*, *Dermacentor*, *Rhipicephalus* and *Amblyomma* frequently reported as vectors of *Anaplasma* in various regions worldwide [16]. Ixodidae ticks can transmit *Anaplasma* species through transstadial transmission, although, with a few exceptions, they do not do so transovarially. Furthermore, *Anaplasma* species have also been discovered in various stages of mosquito development, including eggs, larvae, pupae, and adults [10]. Nevertheless, the role of mosquitoes in the transmission of *Anaplasma* species needs further investigation.

The clinical symptoms of the *Anaplasma* infection range from subclinical to severe, with signs depending on the species involved, bacterial load, and host factors, such as susceptibility, immunity and age [16,17,18]. In general, *Anaplasma* infection in mammals occurs as a subclinical infection or co-infection, and the clinical symptoms may include fever, anemia, ataxia, anorexia, jaundice, or lymph node enlargement [19].

The clinical diagnosis of *Anaplasma* infections is challenging due to non-specific clinical signs. During the acute phase, diagnosis is typically based on the microscopic examination of blood smears stained with Giemsa to detect *Anaplasma* organisms [20]. Although this method is inexpensive and fast, it lacks sensitivity and relies heavily on the skill of the examiner.

To detect pathogen DNA, molecular techniques, such as loop-mediated isothermal amplification (LAMP), polymerase chain reaction (PCR), and real-time quantitative PCR (qPCR), are commonly used. However, the sensitivity of these techniques can be limited, especially in persistently infected animals with low-level bacteremia [21,22]. On the other hand, serological tests, such as the indirect fluorescent antibody technique (IFAT) and the enzyme-linked immunosorbent assay (ELISA), offer advantages for detecting antibodies in infected animals at all stages of the *Anaplasma* infection and for identifying previous exposure to pathogens and carrier animals [1,23,24,25].

Despite the increasing number of studies on *Anaplasma* species in Sardinian ticks [26,27,28,29], knowledge gaps remain regarding the spectrum of these bacterial species among domestic hosts, the potential role of livestock as sources of human infection, and the overall epidemiology of *Anaplasma* in the region. Sardinia is a hotspot for tick-borne infections, characterized by different landscapes, climatic patterns, and vegetation coverage that influence the occurrence of tick-borne diseases. The objective of this study was to investigate the presence and diversity of *Anaplasma* species, including the novel *Anaplasma* species potentially emerging in bovines, horses, and swine in Sardinia, Italy.

## 2. Materials and Methods

### 2.1. Ethical Statement

This study adhered to ethical guidelines and regulations and did not involve any experimental procedures on animals. Sample collection was conducted during routine clinical visits to farms as part of standard veterinary care or disease monitoring.

### 2.2. Study Area and Design

From January 2019 to December 2020, a study was conducted to assess the prevalence of *Anaplasma* species in domestic mammals across various farms on the island of Sardinia, Italy. As part of this study, EDTA blood samples were collected by qualified veterinarians from a total of 158 asymptomatic domestic mammals, including 80 horses, 72 cattle, and 6 pigs. These animals were selected from 49 farming locations across the island, representing a broad cross-section of Sardinia’s agricultural landscape.

All procedures adhered to European Union regulations on animal health and welfare, ensuring that the sample collection did not disrupt animal care or management practices. In compliance with ethical standards, informed consent was obtained from the animal owners, who were thoroughly briefed on the study’s objectives, the sampling process, and any potential risks. The consent process was conducted transparently, and the owners were assured that their animals’ health and well-being would remain a priority throughout the study.

Sardinia’s Mediterranean climate plays a crucial role in the local ecology of ticks and tick-borne diseases. With mild, rainy winters and hot, dry summers, coupled with an average annual temperature of approximately 25 °C, the island provides an ideal environment for tick survival and proliferation. The average annual rainfall of around 200 mm further supports the necessary humidity for tick populations. These climatic conditions create ideal circumstances for the spread of tick-borne pathogens [30]. Consequently, Sardinia has a high incidence of tick-borne diseases, making it a key location for studies of vector-borne pathogens [31]. All the animals sampled in this study were treated for ticks at the beginning of the tick activity period.

### 2.3. DNA Extraction and PCR Strategies

Genomic DNA was extracted from the collected blood samples using the commercially available DNeasy^®^ Blood & Tissue kit (Qiagen, Hilden, Germany), following the manufacturer’s instructions. In our study, all 158 animals were initially screened for *Anaplasma* spp. by PCR amplification using the primers HGE-521F and HGE-790R, which amplify a fragment of the 16S ribosomal RNA gene conserved across all *Anaplasma* species. The PCRs were performed as previously described [32]. Following the initial screening, only the samples that tested positive for *Anaplasma* spp. underwent a second round of testing to further identify and differentiate the *Anaplasma* species using more specific genetic markers. The presence of the species related to *A. platys* and *A. phagocytophilum* was assessed through various PCR assays targeting the *gltA* and *groEL* genes (Table 1). Each PCR included both positive and negative controls. By focusing on the animals that initially tested positive, we ensured that the follow-up PCR assays were performed on individuals with confirmed *Anaplasma* infections, thereby increasing the likelihood of obtaining clear and specific species-level identifications.

The PCR products were examined using 1% agarose gels containing SYBR Safe DNA Gel Stain (Invitrogen, Carlsbad, CA, USA) and visualized under UV light. The gels were then stored at −20 °C for further analysis.

### 2.4. Purification, Sequencing and Phylogenetic Analyses

The PCR products were purified using the QIAquick Purification Kit (Qiagen, Hilden, Germany), following the manufacturer’s instructions. The purified amplicons were then sequenced, assembled, and analyzed using the ChromasPro software (version 1.7.7, Technelysium Pty. Ltd., Tewantin, Australia). The resulting sequences were assigned to unique sequence types, which were named after the host and identified by progressive numbers. These sequence types were compared with entries in the GenBank database using the standard nucleotide Basic Local Alignment Search Tool (BLASTn version 2.16.0; https://blast.ncbi.nlm.nih.gov, accessed on 1 September 2022). All the sequences obtained in this study were deposited in the GenBank database (accession numbers are shown in Table 2).

For the phylogenetic analyses, the four ***gltA*** sequence types (C3–C6) were aligned with a set of nine sequences representing the ***gltA*** variability of the *Ca.* A. cinensis (MH716424, LC645225), *Candidatus* Anaplasma africanum (LC592667, OP654651), *Candidatus* Anaplasma turritanum (MW296131, MW296123, MW296140, OP573281), and *A. platys* (AY077620). The *Rickettsia rickettsii* (MT958042) sequence was used as an outgroup.

The three ***groEL*** sequence types (C7–C9) related to the *A. platys*-like strains were aligned with a set of ten sequences representing the ***groEL*** gene variability of the *Ca.* A. cinensis (MH716434, MH716430, MH716429), *Ca.* Anaplasma africanum (MH610097, KX650588, KX650589, OP573278, OP573279), *Ca.* A. turritanum (OP573342), and *A. platys* (AY848753). The *R. rickettsii* (U96733) sequence was used as an outgroup.

The two ***groEL*** sequence types (C10, H2) related to *A. phagocytophilum* were aligned with a set of ten sequences representing ***groEL*** gene variability of the *A. phagocytophilum* like-1 (MN431807, MN431806, MN431828, JN055360), *A. phagocytophilum* (APHH01000001, CP015376), *Ca.* A. boleense (OP573322, OP573323, KJ410301, OL691654). The *R. rickettsii* (U96733) sequence was used as an outgroup.

The evolutionary history for all the phylogenetic trees was inferred by using the maximum likelihood method. The Kimura 2-parameter model was applied to the ***gltA*** tree and the *A. phagocytophilum **groEL*** tree, while the Tamura–Nei model was used for the *A. platys*-like ***groEL*** tree. The trees with the highest log likelihood (−2331.22, −1767.97, −2710.66, respectively) are shown. The percentage of trees in which the associated taxa clustered together is shown next to the branches. The initial trees for the heuristic search were obtained automatically by applying the neighbor-joining and BioNJ algorithms to a matrix of pairwise distances estimated using the maximum composite likelihood (MCL) approach, and the Tamura–Nei model, respectively. The topology with the superior log likelihood value was then selected. A discrete Gamma distribution was used to model the evolutionary rate differences among the sites (five categories (+*G*, parameter = 1.1311; +G, parameter = 0.2919; +G, parameter = 0.5544, respectively)). These analyses involved 14, 14 and 13 nucleotide sequences, respectively. There was a total of 486, 468, and 719 positions in the final dataset. The evolutionary analyses were conducted in MEGA12 [35].

## 3. Results

*Anaplasma* DNA was detected in 56 out of 158 mammals (35.4%) in this study, including 13/80 horses (16.3%), 39/72 cattle (54.1%), and 4/6 swine (66.7%), using standard PCR targeting the 16S rRNA gene.

The sequencing of the 16S rRNA PCR products generated clear electropherograms in 23 of the 56 positive samples. The sequence alignment revealed four distinct sequence types (Table 2).

The GenBank BLAST analysis showed that ten sequences (HCS1; PQ579998–PQ580007) from two horses, five cattle, and three swine were 100% identical to the *A. capra*/*A. ovis*/*A. centrale*/*A. marginale* group isolated in mammals and ticks worldwide. Although the 16S rRNA gene is highly conserved across the *Anaplasma* species, it was unable to differentiate between these closely related species. Additionally, two sequences from two cattle (C1; PQ580008–PQ580009) were 100% identical to *A. platys* and the related strains. Similarities in the *A. phagocytophilum*/*A. platys/Ca.* A. cinensis group varied from 98.78% in the H1 (PQ580014) sequence isolated from one horse to 100% in the C2 sequence type from four cattle (PQ580010–PQ580013) (Table 2). To achieve greater species-level resolution, these positive samples were subjected to a second round of testing using more specific genetic markers.

PCR testing of the *gltA* gene, which targets the *Anaplasma* sp. related to *A. platys* and *A. capra* strains, combined with the sequencing and BLAST analysis (Table 2), identified three sequence types in four cattle, showing 99.47% to 99.56% similarity to *Ca.* A. cinensis (C3, PQ609681–PQ609682; C4, PQ609683; and C5, PQ609684). Additionally, one sequence type was detected in a single cattle, exhibiting 99.47% similarity to *Ca.* A. turritanum (C6, PQ609685).

The sequencing and BLAST analysis of the three cattle that tested positive for the *A. platys*-like ***groEL*** gene revealed two sequence types (C7 and C8, PQ588703–PQ588704) with 99.22% to 99.32% similarity to *Ca.* A. cinensis and one sequence type (C9, PQ588706) with 92.48% similarity to the *A. platys* gene.

Finally, one cattle and one horse positive for the ***groEL*** gene specific to the *A. phagocytophilum*-like 2 (Table 2) showed 99.47% (C10, PQ588707) and 97.74% (H2, PQ588708) similarity to the *Anaplasma* sp. BL099-6 found in ticks in China.

**Figure 1:**
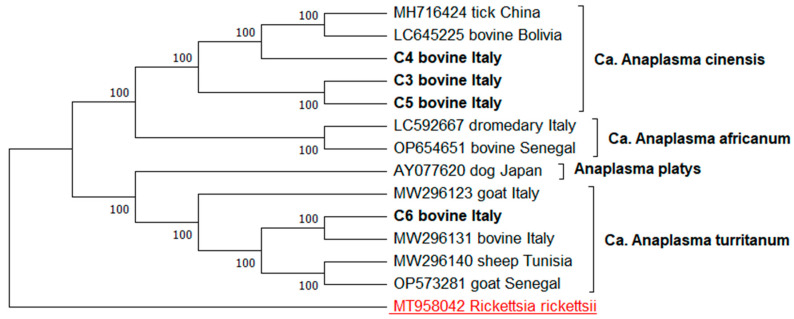
Phylogenetic tree of the *Anaplasma* spp. strains based on ***gltA*** sequencing. Evolutionary history was inferred using the maximum likelihood method and Tamura–Nei model.

The phylogenetic analysis, based on the alignment of the four ***gltA*** gene sequence types obtained in this study with the nine reference *Anaplasma* sequences, revealed four main clades strongly supported by a bootstrap analysis: (a) one clade, including C3, C4 and C5 sequence strains, which clustered with the *Ca.* A. cinensis reference sequences; (b) one clade containing the C6 sequence strains and reference sequences of *Ca.* A. turritanum; (c) one clade containing *A. platys*; (d) one clade containing *Ca.* A. africanum. The C3–C5 sequence types formed a well-supported monophyletic group with *Ca.* A. cinensis reference sequences, which were also identified in ticks from China and bovines from Bolivia. The C6 sequence type grouped with the *Ca.* A. turritanum reference strains, which represent the strains previously reported in ruminants from Italy, Tunisia, and Senegal (Figure 1).

**Figure 2:**
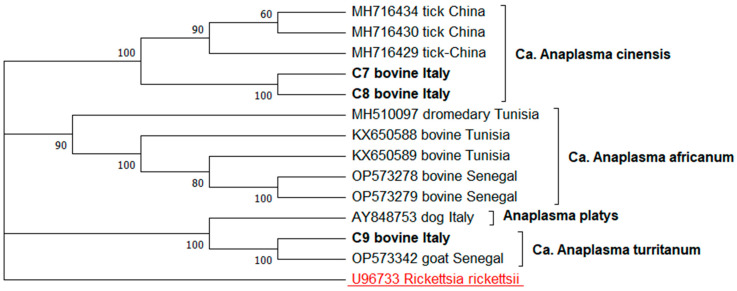
Phylogenetic analysis of the *Anaplasma* spp. strains based on ***groEL*** sequencing. The evolutionary history was inferred using the maximum likelihood method with the Tamura–Nei model.

The phylogenetic tree based on the alignment of the three sequence types named C7, C8 and C9, with 11 reference *Anaplasma* sequence strains, was consistent with the BLASTN results. The sequence types C7 and C8 formed a statistically supported monophyletic clade with the *Ca.* A. cinensis ***groEL*** sequences isolated from ticks in China. Meanwhile, the C9 sequence type clustered in a statistically supported clade with the *Ca*. A. turritanum ***groEL*** gene sequences isolated from dogs in Italy (Figure 2).

**Figure 3:**
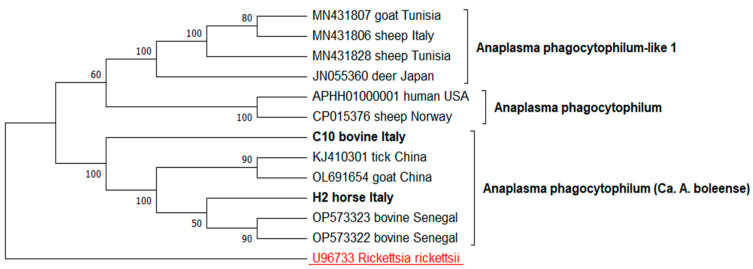
Phylogeny of *A. phagocytophilum* strains based on ***groEL*** sequencing. The evolutionary history was inferred using the maximum likelihood method, applying the Tamura–Nei model.

Finally, the phylogenetic tree constructed from the alignment of the C10 and H2 sequence types with 11 reference *Anaplasma* sequence strains, was consistent with the BLASTN results. Both sequence types were resolved into a monophyletic clade, strongly supported by a bootstrap value of 100%, and grouped with Chinese *A. phagocytophilum*-like 2 sequences (Figure 3).

## 4. Discussion

To date, several *Anaplasma* species have been detected in the Mediterranean area, including the zoonotic *A. phagocytophilum* (found in humans, ticks, dogs, sheep, and horses), *A. platys* (in dogs and ticks), and *A. ovis* (in ticks, sheep, and goats). Additionally, *A. marginale* (in calves and goats), *A. bovis* (in sheep), and *Ca. A. turritanum* have been reported in the same area, including Sardinia [15,26,36,37,38,39].

The molecular approach applied in this study enabled the identification of novel sequence types linked to *A. phagocytophilum* and *A. platys*, while also confirming the presence of *Ca.* A. turritanum, a species commonly found in Mediterranean countries [28]. This species is primarily transmitted through the animal trade and the passive transport of ticks by migratory birds, but it has recently emerged in sub-Saharan Africa, possibly due to the founder effect [40].

The identification and characterization of the *Ca.* A. turritanum strains using the ***gltA*** and ***groEL*** gene markers underscores the value of these genes in the genetic studies of the *Anaplasma* species related to *A. phagocytophilum* and *A. platys* [41]. Detecting genetic variations is crucial for understanding the epidemiology of the emerging *Anaplasma* species, their transmission dynamics, and their potential zoonotic role, which could help develop more targeted, region-specific control strategies, thereby reducing the risk of outbreaks in both livestock and human populations.

These results contribute to understanding the geographic spread of *Ca.* A. turritanum and emphasize the need for continuous surveillance. Further research should focus on tracking its movement between regions, identifying primary and alternative reservoirs and vectors, and evaluating its role in zoonotic transmission. Also, *Ca.* A. turritanum should be included in the diagnostic routine of tick-borne infections in humans to assess its possible impact as a zoonotic agent.

The detection of *Ca. A. cinensis* in Sardinian cattle provides new insights into the geographic distribution of this species. Three distinct sequence types of *Ca.* A. cinensis were identified in cattle, a species genetically related to *A. platys* [40], confirming its presence in Sardinia for the first time in Europe. Initially isolated in 2019 from *Rhipicephalus microplus* ticks in Ankang, Northwest China [33], the presence of *Ca.* A. cinensis in Sardinia constitutes a novel record for Europe. This finding not only expands the understanding of the species’ geographic range but also highlights the potential epidemiological trends common to other Mediterranean countries where similar arthropod vectors are prevalent.

Another significant discovery is the first detection in Sardinia of *A. phagocytophilum*-like 2 (the Chinese variant of *A. phagocytophilum*) in one horse and one cattle. This strain, previously described in China in mosquitoes (*Aedus albopictus*, *Anopheles sinensis*, and *Armigeres subalbatus*) at various life stages, had been classified as *Ca.* A. boleense [10]. This finding underscores the need for further research to understand its transmission pathways and the potential impact on animal and human health.

Currently, *Ca. A. turritanum*, *Ca. A. cinensis*, and *Ca.* A. boleense are not included in standard diagnostic panels for tick-borne infections of human and veterinary significance, despite evidence of their presence in domestic mammals across various Mediterranean regions. Furthermore, none of the animals analyzed in this study exhibited the clinical signs of illness, which supports the hypothesis that ruminants and equines may act as reservoirs for these *Anaplasma* species. The choice of asymptomatic animals was intentional. By selecting animals that were apparently healthy at the time of sampling, this study aimed to assess the presence of latent or subclinical infections, which are common in tick-borne diseases but often go undetected without targeted testing. This approach is crucial for understanding the true prevalence of infections in livestock, as many tick-borne pathogens can persist in animals without causing overt symptoms. In this way, the study aimed to provide a more accurate picture of the circulation of tick-borne pathogens in the region, beyond the clinical cases that might be detected only during outbreaks.

The impact of *Ca. A. turritanum*, *Ca.* A. cinensis, and *Ca.* A. boleense on human health requires further investigation, as these species may represent emerging zoonotic threats. The naming of variants as *Candidatus* is crucial for understanding the scientific, diagnostic, pathogenic, and epidemiological implications. Taxonomically, *Candidatus* indicates species that have been identified but not fully described, creating diagnostic challenges due to genetic variations that can lead to cross-reactions in tests. These genetic differences can influence the pathogenicity, immune response, and treatment effectiveness. Epidemiologically, the classification highlights the need for further research on the distribution and spread of these species, as even slight genetic variations can affect the transmission and impact on populations, with implications for public health strategies and zoonotic disease monitoring.

As the geographic range of *Anaplasma* species continues to expand, there is an increasing need for enhanced surveillance systems to able to detect and to monitor these pathogens across regions. Additionally, improving diagnostic strategies for both humans and animals to enable the early detection of these pathogens is key to preventing severe disease outcomes. Molecular-based diagnostics, including PCR and sequencing technologies, have proven invaluable in accurately identifying *Anaplasma* species and their genetic variants, facilitating timely interventions and treatment.

## 5. Conclusions

The identification of *Ca.* A. turritanum, *Ca.* A. cinensis, and *Ca.* A. boleense in domestic mammals reveals that these species, closely related to the zoonotic *A. platys* and *A. phagocytophilum*, are emerging. Climate-related changes may be facilitating their spread by altering tick population dynamics and expanding the distribution of vector species. This highlights the urgent need to include these emerging *Anaplasma* species in routine diagnostic practices for both humans and animals, as they could represent a significant threat to public and veterinary health. Effective surveillance and control measures are crucial to mitigate the potential spread of these pathogens and to reduce the risk of the zoonotic transmission of *Anaplasma* species. Further research is needed to fully understand the epidemiology of these species and refine strategies for their detection, prevention, and control.

## Figures and Tables

**Table 1:** Primer sequences used in this study for the detection of *Anaplasma* species from the blood samples of the mammal hosts analyzed.

Pathogens	Gene Target	PCR Assay	Gene Primers	Primer Sequence (5′ to 3′)	Amplicon Size (bp)	References
*Anaplasma* spp.	16S rRNA	PCR	HGE–521F HGE–790R	TGTAGGCGGTTCGGTAAGTTAAAG	293 bp	[32]
				CTTAACGCGTTAGCTACAACACAG		
*A. platys*	*glt*A	Nested PCR	Pglt-F	ATGAWAGAAAAWGCTGTTTT	660 bp	[33]
			Pglt-R1	TCATGRTCTGCATGCATKATG		
			Pglt-R2	CATGCATKATGAARATMGCAT		
			Pglt-L-F1	ATGAWAGAAAAWGCTGTTTT	610 bp	
			Pglt-L-F2	TCATGRTCTGCATGCATKATG		
			Pglt-L-R	CATGCATKATGAARATMGCAT		
	GroEL	Nested PCR	Pgro_F_F	GATGCWCATCCYATSGCMATG	1195 bp	
			Pgro_F_R1	CGTGMTSGCTATAGCGMAART		
			Pgro_F_R2	TCAYACCATTGDGAYRCCCAT		
*A. phagocytophilum*	GroEL	Nested PCR	EphplGroEL(569)F	ATGGTATGCAGTTTGATCGC	573 bp	[26]
			EphplGroEL(1193)R	TCTACTCTGTCTTTGCGTTC		
			EphGroEL(1142)R	TTGAGTACAGCAACACCACCGGAA		
*A. phagocytophilum*-like 1	GroEL	Nested PCR	APGRSP41F3	GAATCTAGCTATGTTGCATGATAATGT	1446 bp	[34]
			APSPGR1697R1	CAGCATAAACACGCACTACGAA		
			APSPGR234F2	CGTAGTAGGACTTTCCGGTTTTTG		
			APSPGR1680R2	TGCACAGCATAAACACGCACTA		
*A. phagocytophilum*-like 2	GroEL	Nested PCR	APHAGOVAR2GROEL_F	TACTCTAGAAGACGCGGTAG	793 bp	[28]
			APHAGOVAR2GROEL_R1	ACGAACATTCTTAGCAGTCC		
			APHAGOVAR2GROEL_R2	CTTCTATCACCAAATCCTGG		

**Table 2:** Definition of 16S rRNA, gltA and groEL gene sequence types, host sources, BLAST analysis and GenBank accession numbers.

Target Gene	Sequence Type *	Host Sources	Locality	BlastN	GenBank Accession Number (s)
16S rRNA	HCS1	Horse, Cattle, Swine	Aglientu, Arzachena, Baunei, Birori, Chiaramonti, Fordongianus, Laerru, Orgosolo, Santu LussurgiuSedini, Villanova Monteleone	100% *A. capra*/*A. ovis*/*A. centrale*/*A. marginale* group	PQ579998–PQ580007
	C1	Cattle	Anela, Chiaramonti, Milis, Oristano, Monti	100% *A. phagocytophilum/A. platys*/*Ca.* A. cinensis group	PQ580008–PQ580009
	C2	Cattle	Orgosolo, Santa Giusta, Villacidro, Laerru, Chiaramonti	100% *A. phagocytophilum/A. platys*/*Ca.* A. cinensis group	PQ580010–PQ580013
	H1	Horse	Santa Giusta	98.78% *A. phagocytophilum/A. platys*/*Ca.* A. cinensis group	PQ580014
*glt*A	C3	Cattle	Orgosolo	99.56% *Ca.* A. cinensis	PQ609681–PQ609682
	C4	Cattle	Orgosolo	99.47% *Ca.* A. cinensis	PQ609683
	C5	Cattle	Orgosolo	99.47% *Ca.* A. cinensis	PQ609684
	C6	Cattle	Illorai	99.47% *Ca.* A. turritanum	PQ609685
*GroEL*	C7	Cattle	Orgosolo	99.32% *Ca* A. cinensis	PQ588703–PQ588704
	C8	Cattle	Orgosolo	99.22% *Ca.* A. cinensis	PQ588705
	C9	Cattle	Milis	99–100% *Ca.* A. turritanum	PQ588706
*GroEL*	C10	Cattle	Bortigiadas	97.74% *Anaplasma* sp	PQ588707
	H2	Horse	Santa Giusta	99.47% *Anaplasma* sp.	PQ588708

* Hosts are numbered according to the order in which they were collected and processed in the laboratory.

## Data Availability

All data generated and analyzed during this study are included in this published article.
